# Cardiovascular Disease Risk with Integrase Inhibitor-based Antiretroviral Therapy: A Causal Inference Analysis in RESPOND

**DOI:** 10.1093/ofid/ofag490

**Published:** 2026-08-06

**Authors:** Bernard Surial, Bastian Neesgaard, Frédérique Chammartin, Lene Ryom, Kathy Petoumenos, Joanne Reekie, Roger Paredes, Robert Zangerle, Colette Smith, Charlotte Martin, Ferdinand Wit, Nadine Jaschinski, Cristina Mussini, Antonella d’Arminio Monforte, Antonella Castagna, Christina Carlander, Larysa Hetman, Elena Sohani, Clara Lehmann, Sean R Hosein, Jean Andre Van Wyk, Emiliano Bissio, Jens Lundgren, Andri Rauch, Huldrych F Günthard, Gilles Wandeler, Lauren Greenberg

**Affiliations:** Department of Infectious Diseases, Inselspital, Bern University Hospital, University of Bern, Bern, Switzerland; CHIP, Centre of Excellence for Health, Immunity, and Infections, Rigshospitalet, University of Copenhagen, Copenhagen, Denmark; Division of Clinical Epidemiology, Department of Clinical Research, University Hospital and University of Basel, Basel, Switzerland; Department of Infectious Diseases, Inselspital, Bern University Hospital, University of Bern, Bern, Switzerland; Department of Infectious Diseases 144, Hvidovre University Hospital, Copenhagen, Denmark; Department of Clinical Medicine, University of Copenhagen, Copenhagen, Denmark; The Kirby Institute, UNSW Sydney, Sydney, New South Wales, Australia; CHIP, Centre of Excellence for Health, Immunity, and Infections, Rigshospitalet, University of Copenhagen, Copenhagen, Denmark; Department of Infectious Diseases, Hospital Universitari Germans Trias I Pujol, Badalona, Spain; Department of Dermatology, Venereology, and Allergy, Medical University Innsbruck, Innsbruck, Austria; The Royal Free HIV Cohort Study, Royal Free Hospital, University College London, London, UK; Infectious Diseases Department, CHU Saint-Pierre, Université Libre de Bruxelles (ULB), Brussels, Belgium; AIDS Therapy Evaluation in the Netherlands (ATHENA) Cohort, HIV Monitoring Foundation, Amsterdam, The Netherlands; CHIP, Centre of Excellence for Health, Immunity, and Infections, Rigshospitalet, University of Copenhagen, Copenhagen, Denmark; Modena HIV Cohort, Università degli Studi di Modena, Modena, Italy; Icona Foundation, Milan, Italy; Icona Foundation, Milan, Italy; Department of Infectious Diseases, Karolinska University Hospital, Stockholm, Sweden; Public Health Center of Ukraine, Ministry of Health, Kyiv, Kyiv Oblast, Ukraine; Department of Dermatology, Venereology, and Allergy, Medical University Innsbruck, Innsbruck, Austria; Department I of Internal Medicine, Division of Infectious Diseases, Medical Faculty and University Hospital Cologne, University of Cologne, Cologne, Germany; German Center for Infection Research, Partner Site Bonn-Cologne, Cologne, Germany; European AIDS Treatment Group, Brussels, Belgium; ViiV Healthcare, London, UK; Merck & Co., Inc., Rahway, New Jersey, USA; CHIP, Centre of Excellence for Health, Immunity, and Infections, Rigshospitalet, University of Copenhagen, Copenhagen, Denmark; Department of Infectious Diseases, Inselspital, Bern University Hospital, University of Bern, Bern, Switzerland; Department of Infectious Diseases and Hospital Epidemiology, University Hospital Zurich, University of Zurich, Zurich, Switzerland; Institute of Medical Virology, University of Zurich, Zurich, Switzerland; Department of Infectious Diseases, Inselspital, Bern University Hospital, University of Bern, Bern, Switzerland; Institute of Social and Preventive Medicine, University of Bern, Bern, Switzerland; CHIP, Centre of Excellence for Health, Immunity, and Infections, Rigshospitalet, University of Copenhagen, Copenhagen, Denmark

**Keywords:** cardiovascular disease, HIV, integrase inhibitors, target trial emulation

## Abstract

**Background:**

A previous RESPOND analysis suggested that integrase inhibitors (INSTIs) increase cardiovascular disease (CVD) risk compared with other antiretroviral therapy (ART). We reanalyzed the data using target trial emulations examining the impact of methodological choices.

**Methods:**

Treatment-naïve analyses included ART-naïve adults with detectable HIV viraemia and recent CD4 and HIV-RNA measurements. Treatment-experienced analyses included adults with suppressed HIV-RNA receiving INSTI-free ART. We estimated adjusted risk differences (RDs) and risk ratios (RRs) for CVD events comparing INSTI initiation or switching versus other ART using inverse-probability weighting and pooled logistic regression.

**Results:**

Among 7111 treatment-naïve individuals, 95 CVD events occurred over a median of 62 months. Risk ratios comparing INSTI initiation with starting other ART were 1.07 (95% confidence interval .29–4.93) at 1 year, 1.21 (.41–3.91) at 2 years, and 1.87 (1.06–3.26) at 6 years. Corresponding RDs were 0.02% (−.32–.29), 0.09% (−.43–.53), and 1.03% (0.08–2.07). Among 22 921 treatment-experienced individuals, 800 CVD events occurred over a median of 37 months. Risk ratios were 1.55 (1.16–2.00) at 1 year, 1.30 (1.07–1.62) at 2 years, and 0.93 (.78–1.09) at 6 years. Corresponding RDs were 0.26% (.08–.47), 0.29% (.03–.58), and −0.22 (−.66–.25).

**Conclusions:**

Switching to INSTIs was associated with a transient increase in CVD risk during the first 2 years among treatment-experienced individuals, whereas starting INSTIs in ART-naïve individuals was associated with a gradual increase over time, albeit based on few events. Absolute RDs remained small, but the findings suggest a potential impact of INSTIs on the development of CVD.

## Teaser

In these target trial emulations in RESPOND, integrase inhibitor (INSTI)-based antiretroviral therapy was associated with a transiently increased cardiovascular disease risk among treatment-experienced people with HIV and a gradual risk increase in treatment-naïve individuals.

People with HIV-1 face a substantially higher cardiovascular disease (CVD) risk than the general population, reflecting both a higher prevalence of CVD risk factors and HIV-related chronic immune activation accelerating atherosclerosis [1–3]. Adverse effects of antiretroviral therapy (ART) may also contribute, as observed with certain protease inhibitors (PIs) and abacavir (ABC) [4–11].

In the International Cohort Consortium of Infectious Diseases (RESPOND), our post-marketing pharmacovigilance efforts identified an increased CVD risk during the first 2 years of integrase strand transfer inhibitor (INSTI) exposure compared with non-INSTI-based ART [12]. This rapid risk increase was inconsistent with the notion that atherosclerosis requires longer to develop [13].

Pharmacovigilance requires multiple complementary studies to confirm safety signals and explore mechanisms. Further studies have been called for to investigate these findings related to INSTIs in independent cohorts and through alternative study designs. The target trial framework applies design principles of randomized trials to reduce potential bias in observational studies by aligning start of follow-up, participant eligibility, and treatment assignment [14]. This is particularly important when comparing individuals who switch ART with those remaining on other ART who lack a natural baseline.

While some cohort studies also found an increased CVD risk with use of INSTIs, these findings were not confirmed by two target trial emulations [15, 16]. First, the Swiss HIV Cohort Study (SHCS) compared CVD events between treatment-naïve individuals starting INSTI-based ART with those starting other ART [17]. Second, the Antiretroviral Therapy Cohort Collaboration (ART-CC) and HIV-CAUSAL Collaboration assessed CVD risk in ART-naïve and experienced individuals [18]. Both studies reported no increase in CVD risk with the use of INSTIs compared to other ART.

In this study, we performed a target trial emulation to estimate the impact of INSTI-based ART on CVD risk among treatment-naïve and treatment-experienced individuals with HIV in RESPOND.

## METHODS

### Data Source

RESPOND is a collaboration of 19 cohorts of people with HIV across Europe and Australia [19]. Clinical and laboratory data are prospectively recorded within each local cohort during routine practice and shared annually with RESPOND using standardized protocols. All study sites' ethical committees approved the cohort studies, and all participants provided consent according to local regulations. Findings are reported following the Strengthening the Reporting of Observational Studies in Epidemiology guidelines [20]. Community representatives contributed to the study planning, interpretation of results, and manuscript preparation.

### Eligibility Criteria and Treatment Strategies

We emulated two target trials: (1) among treatment-naïve individuals initiating ART and (2) among ART-experienced individuals with a suppressed HIV viral load (Supplementary Tables 1**/**2). Treatment-naïve individuals had to be ≥18 years and ART-naïve after January 2012 (when INSTIs became widely available in Europe) and have a detectable HIV viral load (≥200 cp/mL) and CD4 and viral load measurements within 1 year before or up to 12 weeks after ART start. Individuals with missing data on gender/sex, who started unknown ART or who started maraviroc or enfuvirtide (unusual initial ART regimens), were excluded. Eligibility criteria for treatment-experienced individuals were similar, except individuals needed to be on INSTI-free ART after January 2012 without prior exposure to INSTIs and have a suppressed HIV viral load (<200 cp/mL). Individuals with a history of CVD were excluded in the treatment-experienced analysis.

In treatment-naïve analyses, individuals were classified as initiating either an INSTI-based ART regimen—regardless of additional components such as non-nucleoside reverse transcriptase inhibitors (NNRTIs) or PIs—or ART without INSTI. In the treatment-experienced analyses, individuals followed one of two strategies: switching to an INSTI-based or continuing INSTI-free regimen. Within the INSTI-free strategy, participants were permitted to switch ART provided none contained an INSTI. Similarly, individuals following the INSTI-based strategy could switch between different INSTIs.

### Follow-up and Outcomes

Baseline was defined as the start date of the initial ART regimen for treatment-naïve individuals. In the treatment-experienced analyses, a new trial was emulated each month between January 2012 and December 2021. Baseline covariates were redefined and eligibility reassessed at each trial start, meaning individuals could contribute to multiple nested trials over time. Treatment assignment was determined according to the ART regimen at baseline of each trial start, without applying a post-baseline grace period. These overlapping sequential trials were subsequently pooled for analysis (Supplementary Figure 1, Supplementary note).

In the intention-to-treat (ITT) analyses, participants were followed from baseline until earliest of CVD event, death, loss to follow-up, or final cohort visit. Individuals without HIV viral load measurement for ≥18 months were considered lost to follow-up. In per-protocol (PP) analyses, participants were artificially censored when they deviated from their assigned treatment strategy (eg, switching from INSTI-based to non-INSTI ART or starting INSTI while assigned to continue non-INSTI ART).

The outcome of interest was time to first CVD event, including fatal and nonfatal myocardial infarctions, strokes, and invasive cardiovascular procedures (coronary angioplasty or stenting, coronary bypass surgery, and carotid artery stenting or endarterectomy) [21]. All CVD events occurring within 1 year before or any time after RESPOND enrollment were reported using standardized case report forms and centrally validated by a trained physician using established algorithms [22]. Earlier events were recorded but not centrally validated.

### Statistical Analysis

The statistical approach was similar for both treatment-naïve and treatment-experienced populations. For ITT analyses, we used pooled logistic regression to estimate risk differences and risk ratios between exposure categories (ART with vs without INSTI) on CVD event risk. Models included linear and quadratic product terms between time and exposure. To adjust for confounding, we used stabilized inverse probability of treatment weights, estimated using pooled logistic regression. The treatment-naïve weight model included the following baseline covariates: age, gender/sex, race/ethnicity (White, Black, other), local cohort, HIV transmission risk (men who have sex with men [MSM], heterosexual contact, persons who inject drugs, other), nadir and baseline CD4 cell counts (≥500, 350–499, 200–349, <200 cells/µL), HIV viral load (50–199, 200–100'000, >100'000 cp/mL), calendar year of ART start (before 2015, 2015–2016, after 2016), history of AIDS-defining illness, body mass index (BMI; <18.5, 18.5–24.9, 25–29.9, ≥30 kg/m^2^), diabetes, arterial hypertension, estimated glomerular filtration rate (eGFR; ≥90, 60–89, <60 mL/min, calculated with the Chronic Kidney Disease-Epidemiology Collaboration formula [23]), history of CVD, smoking status (never, previous, current), total cholesterol (<4.1, 4.1–5.19, 5.2–6.19, 6.2–7.19, ≥7.2 mmol/L), high density lipoprotein (HDL; <0.9, 0.9–1.19, 1.2–1.29, 1.3–1.59, ≥1.6 mmol/L), and whether initial ART included tenofovir alafenamide (TAF) or ABC. Diabetes was defined as a random blood glucose >11.1 mmol/L, a glycated hemoglobin concentration of >48 mmol/mol (>6.5%), use of antidiabetic drugs, or a clinical diagnosis of diabetes reported by the local cohort. Arterial hypertension was defined as systolic blood pressure >140 mmHg, diastolic blood pressure >90 mmHg, or use of antihypertensive drugs. We used the same covariates for the treatment-experienced analyses, except that we removed history of CVD (these individuals were not eligible) and baseline HIV viral load (a suppressed HIV viral load was required) and added year of ART start and an indicator for each nested trial as linear and quadratic terms.

Models for PP analyses were similar; however, individuals were artificially censored when they deviated from their assigned strategy. In the treatment-naïve PP analysis, we additionally fitted a pooled logistic regression model to calculate inverse probability of censoring weights to adjust for potential selection bias introduced through artificial censoring. This model included baseline values of age, gender/sex, race/ethnicity, local cohort, HIV transmission risk, nadir CD4, year of ART start, history of AIDS-defining illness, history of CVD, and time-varying values for CD4, HIV viral load, BMI, diabetes, arterial hypertension, eGFR, smoking status, total cholesterol, HDL, TAF, and ABC use. The treatment-naïve PP outcome model was weighted by the product of the baseline inverse probability of treatment weights and inverse probability of censoring weights. In the treatment-experienced PP analysis, we used cumulative inverse probability of treatment weights across sequential trials, estimated using the same covariates as in the ITT treatment-experienced weight model and including an indicator for each trial. All weights were truncated at the 99th percentile to limit the influence of extreme weights and used to weight the study population in the outcome model (Supplementary Table 3).

We calculated 95% confidence intervals (CI) using bootstrapping with 500 samples. We did not impute missing data but included a separate indicator for missing information. We used SAS 9.4 for data preparation and R 4.3.3 for statistical analyses.

### Subgroup and Sensitivity Analyses

Because clinical events were few among treatment-naïve individuals, subgroup and sensitivity analyses focused on treatment-experienced individuals. Subgroup analyses were performed by sex/gender (female, male), age (<65, ≥65 years), and whether the baseline ART included ABC. We also performed multiple sensitivity analyses. First, to assess robustness to the choice of causal estimator, we repeated the ITT analyses using parametric g-computation based on pooled logistic regression models adjusted for the baseline covariates listed above, rather than inverse probability of treatment weighting. Second, only sequential trials starting after January 2017 were included, when all RESPOND data were collected prospectively. Third, in cohorts that do not share data on all of their participants with RESPOND, individuals are randomly selected by the cohorts via an algorithm stratified by INSTI use (https://chip.dk/research-areas/cohort-studies/studies-and-projects/respond/study-documents/), which could introduce selection bias. To address this, we restricted the analysis to only include cohorts who share all available data on all their participants with RESPOND; this included four cohorts with 6887 participants.

## RESULTS

### Treatment-naïve Analysis

**Figure 1.**
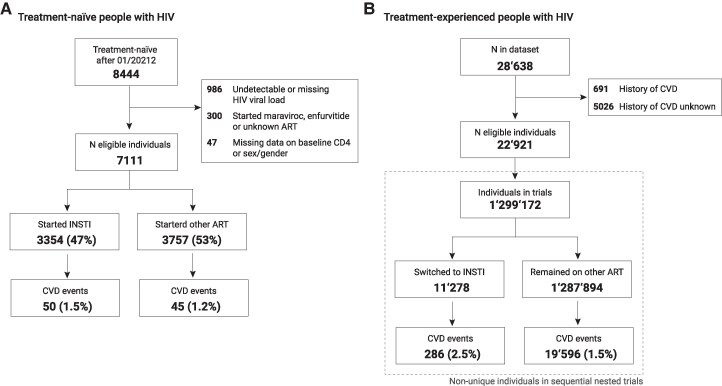
Selection of the study participants. The flow charts show the selection of treatment-naïve (Panel *A*) and treatment-experienced (Panel *B*) individuals with HIV for the target trial emulations. In the treatment-naïve analysis, individuals with a history of CVD were not excluded, but the models were adjusted for this confounder. In the treatment-experienced analysis, individuals were eligible for multiple sequential nested trials. Therefore, the numbers in the *dashed box* represent nonunique individuals. N = number, INSTI = integrase strand transfer inhibitor, ART = antiretroviral therapy, CVD = cardiovascular disease.

Of 8444 individuals in the RESPOND cohort who were treatment-naïve after January 2012, 7111 were included in the analysis (Figure 1*A*). Overall, 1341 (19%) were women, 601 (8.5%) were Black people, the median age at ART start was 38 years (interquartile range [IQR] 31–47), and the main mode of HIV acquisition was MSM (n = 3950, 56%, Table 1).

**Table 1.** Baseline characteristics of the treatment-naïve population with HIV

Characteristic	Started INSTI N = 3354	Started Other ART N = 3757	Overall N = 7111
Female	537 (16%)	804 (21%)	1341 (19%)
Median age, years (IQR)	39 (31–48)	38 (31–46)	38 (31–47)
Ethnicity			
White	2206 (66%)	2581 (69%)	4787 (67%)
Black	260 (7.8%)	341 (9.1%)	601 (8.5%)
Other	237 (7.1%)	174 (4.6%)	411 (5.8%)
Unknown	651 (19%)	661 (18%)	1312 (18%)
Cohort region			
Western Europe	1660 (49%)	1308 (35%)	2968 (42%)
Southern Europe	1014 (30%)	1285 (34%)	2299 (32%)
Northern Europe / Australia	591 (18%)	697 (19%)	1288 (18%)
Eastern Europe	89 (2.7%)	467 (12%)	556 (7.8%)
HIV transmission risk			
MSM	2015 (60%)	1935 (52%)	3950 (56%)
Person who injects drugs	169 (5.0%)	322 (8.6%)	491 (6.9%)
Heterosexual	977 (29%)	1319 (35%)	2296 (32%)
Other	36 (1.1%)	44 (1.2%)	80 (1.1%)
unknown	157 (4.7%)	137 (3.6%)	294 (4.1%)
CD4 cell count			
≥500 cells/µL	1047 (31%)	883 (24%)	1930 (27%)
350–499 cells/µL	740 (22%)	1052 (28%)	1792 (25%)
200–349 cells/µL	687 (20%)	964 (26%)	1651 (23%)
<200 cells/µL	880 (26%)	858 (23%)	1738 (24%)
Unknown	0 (0%)	0 (0%)	0 (0%)
Nadir CD4 cell count			
≥500 cells/µL	903 (27%)	691 (18%)	1594 (22%)
350–499 cells/µL	784 (23%)	1070 (28%)	1854 (26%)
200–349 cells/µL	758 (23%)	1081 (29%)	1839 (26%)
<200 cells/µL	909 (27%)	915 (24%)	1824 (26%)
Unknown	0 (0%)	0 (0%)	0 (0%)
HIV viral load			
50–199 cp/mL	0 (0%)	2 (<0.1%)	2 (<0.1%)
200–100 000 cp/mL	2008 (60%)	2541 (68%)	4549 (64%)
>100 000 cp/mL	1346 (40%)	1214 (32%)	2560 (36%)
Missing	0 (0%)	0 (0%)	0 (0%)
History of AIDS	284 (8.5%)	288 (7.7%)	572 (8.0%)
Initiated ABC-based ART	829 (25%)	477 (13%)	1306 (18%)
Initiated TAF-based ART	923 (28%)	213 (5.7%)	1136 (16%)
Year of ART start			
Before 2015	618 (18%)	2608 (69%)	3226 (45%)
2015–2016	1273 (38%)	765 (20%)	2038 (29%)
After 2016	1463 (44%)	384 (10%)	1847 (26%)
Renal function (eGFR)			
≥90 mL/min	1883 (56%)	2387 (64%)	4270 (60%)
60–89 mL/min	393 (12%)	482 (13%)	875 (12%)
<60 mL/min	36 (1.1%)	32 (0.9%)	68 (1.0%)
Unknown	1042 (31%)	856 (23%)	1898 (27%)
Diabetes	92 (2.7%)	68 (1.8%)	160 (2.3%)
History of CV disease			
No	2363 (70%)	2425 (65%)	4788 (67%)
Yes	16 (0.5%)	14 (0.4%)	30 (0.4%)
Unknown	975 (29%)	1318 (35%)	2293 (32%)
BMI category			
Underweight (<18.5 kg/m^2^)	134 (4.0%)	102 (2.7%)	236 (3.3%)
Normal (18.5–24.9 kg/m^2^)	1450 (43%)	1474 (39%)	2924 (41%)
Overweight (25.0–29.9 kg/m^2^)	556 (17%)	503 (13%)	1059 (15%)
Obese (≥30 kg/m2)	140 (4.2%)	211 (5.6%)	351 (4.9%)
Unknown	1074 (32%)	1467 (39%)	2541 (36%)
Arterial hypertension			
No	2513 (75%)	2905 (77%)	5418 (76%)
Yes	589 (18%)	382 (10%)	971 (14%)
Unknown	252 (7.5%)	470 (13%)	722 (10%)
Smoking status			
Never	975 (29%)	1112 (30%)	2087 (29%)
Current	1245 (37%)	1241 (33%)	2486 (35%)
Previous	183 (5.5%)	215 (5.7%)	398 (5.6%)
Unknown	951 (28%)	1189 (32%)	2140 (30%)
Total cholesterol			
<4.1 mmol/L	1230 (37%)	1243 (33%)	2473 (35%)
4.1–5.19 mmol/L	884 (26%)	1048 (28%)	1932 (27%)
5.2–6.19 mmol/L	262 (7.8%)	309 (8.2%)	571 (8.0%)
6.2–7.19 mmol/l	46 (1.4%)	66 (1.8%)	112 (1.6%)
≥7.2 mmol/L	15 (0.4%)	16 (0.4%)	31 (0.4%)
Unknown	917 (27%)	1075 (29%)	1992 (28%)
HDL			
<0.9 mmol/L	680 (20%)	707 (19%)	1387 (20%)
0.9–1.19 mmol/L	777 (23%)	755 (20%)	1532 (22%)
1.2–1.29 mmol/L	231 (6.9%)	254 (6.8%)	485 (6.8%)
1.3–1.59 mmol/l	285 (8.5%)	310 (8.3%)	595 (8.4%)
≥1.6 mmol/L	215 (6.4%)	228 (6.1%)	443 (6.2%)
Unknown	1166 (35%)	1503 (40%)	2669 (38%)
Receiving lipid-lowering treatment	86 (2.6%)	55 (1.5%)	141 (2.0%)
Receiving antiplatelet drug	55 (1.6%)	55 (1.5%)	110 (1.5%)

IQR = interquartile range, INSTI = integrase strand transfer inhibitor, ART = antiretroviral therapy, N = number, MSM = men who have sex with men, ABC = abacavir, TAF = tenofovir alafenamide, eGFR = estimated glomerular filtration rate, CVD = cardiovascular disease, BMI = body mass index, HDL = high-density lipoprotein.

Compared to individuals who initiated other ART regimens, those starting INSTI-based combinations were more likely to be male, have acquired HIV through MSM contact, and to be from a Western European cohort. Smoking status and total cholesterol were similar between the two groups, but individuals who started INSTI-based ART were more likely to have arterial hypertension, diabetes, and an eGFR <60 mL/min and to start ART with TAF or ABC. Covariate balance before and after inverse-probability weighting is shown in Supplementary Figure 2.

Of individuals who started INSTI-based ART, the majority started dolutegravir (n = 1820, 55.1%), followed by elvitegravir (640, 19.4%), raltegravir (561, 17%), bictegravir (280, 8.5%), and cabotegravir (1, 0.03%). Among those who started other ART, 1977 (52.5%) started NNRTIs, with the most common being efavirenz (n = 948) and rilpivirine (n = 839), and 1744 (46.5%) started boosted PIs, with the most common being darunavir (n = 1145) and atazanavir (n = 433); 36 (1%) participants started a combination of NNRTI and PI.

**Figure 2.**
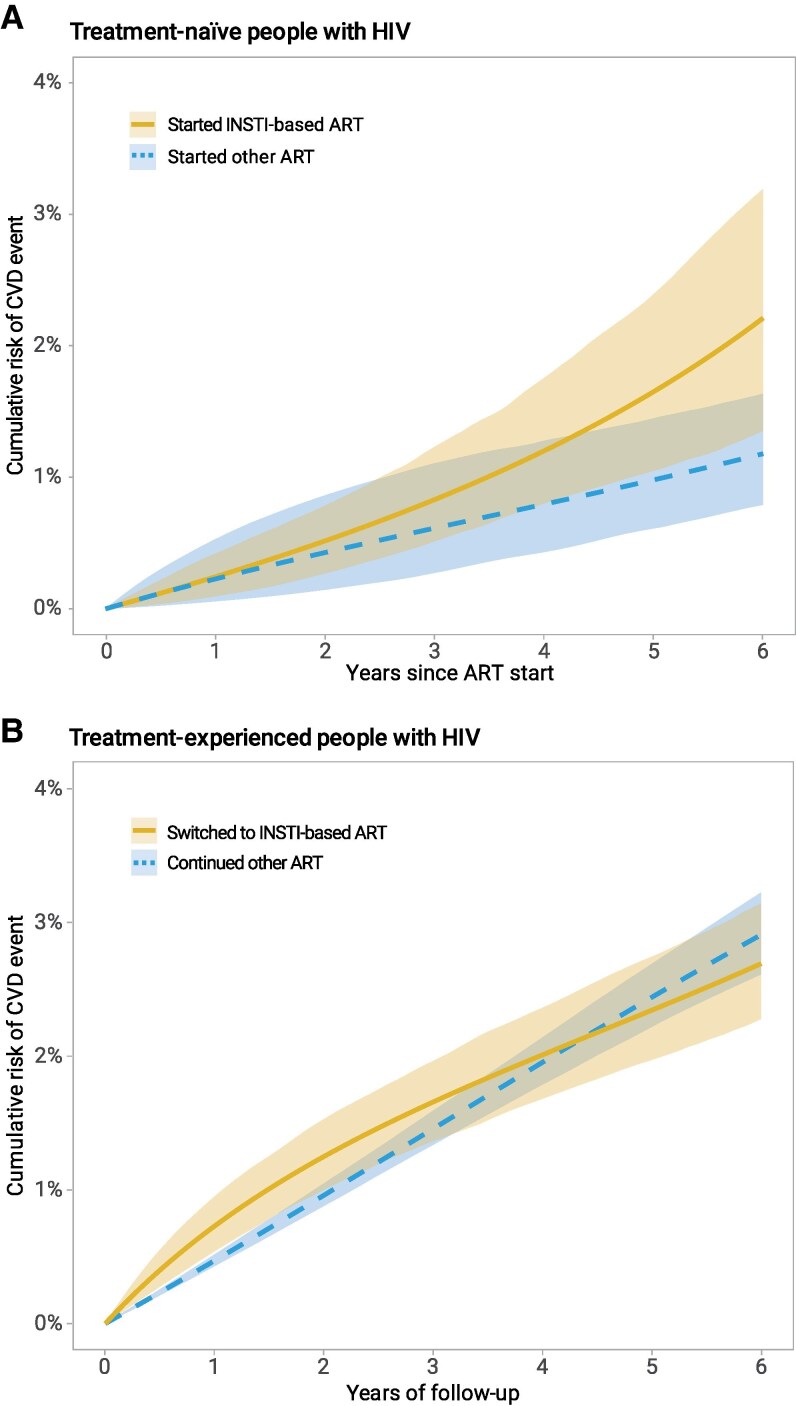
Adjusted cumulative risk of developing cardiovascular disease events. The cumulative risk (*line*) of developing cardiovascular disease (CVD) events and corresponding 95% confidence intervals (*shaded area*) are shown for 7111 treatment-naïve (Panel *A*) and 22 921 treatment-experienced (Panel *B*) people with HIV. CVD events include myocardial infarction, stroke, and procedures on arteries. Results are shown for the intention-to-treat analysis, and confidence intervals were calculated using bootstrapping with 500 samples. INSTI = integrase strand transfer inhibitor, ART = antiretroviral therapy.

During a median follow-up of 62 months (IQR 36–85), 95 CVD events occurred, including 38 acute myocardial infarctions (40%), 37 strokes (39%), and 20 invasive cardiovascular procedures (21%). Of those events, 50 occurred in people who started ART with an INSTI, and 45 in those who started other ART (Supplementary Table 4). In the ITT analysis, the adjusted risk ratio comparing individuals who started an INSTI to those who started other ART was 1.07 (95% CI .20–4.93) at 1 year, 1.21 (95% CI .41–3.91) at 2 years, and 1.87 (95% CI 1.06–3.26) at 6 years. Adjusted risk differences ranged from 0.02 percentage points (95% CI −.32–.29) after 1 year to 1.03 percentage points (95% CI .08–2.07) after 6 years (Table 2, Figure 2*A*). In the PP analysis, CVD risk was consistently higher among individuals who started and remained on INSTI-based ART compared with those who started other ART, reaching an absolute risk difference of 1.30 percentage points (95% CI .37–2.31) after 6 years (Table 2, Supplementary Figure 3A).

**Table 2:** Adjusted risk estimates for cardiovascular disease events, stratified by study population and analysis

Treatment-naïve individuals					
		Risk for cardiovascular disease events			
Analysis	Time (years)	Started INSTI (95% CI)	Started other ART (95% CI)	Risk Difference (95% CI)	Risk Ratio (95% CI)
Intention to treat	1	0.24% (.10–.42)	.23% (.06–.53)	.02% (−.32–.29)	1.07 (.29–4.93)
	2	0.52% (.27–.78)	.43% (.15–.86)	.09% (−.43–.53)	1.21 (.41–3.91)
	4	1.20% (.80–1.75)	.79% (.43–1.28)	.41% (−.26–1.13)	1.51 (.77–3.23)
	6	2.21% (1.36–3.19)	1.18% (.79–1.64)	1.03% (.08–2.07)	1.87 (1.06–3.26)
Per protocol	1	0.22% (.07–.39)	.08% (.01–.15)	.14% (−.01–.32)	2.76 (.84–16.05)
	2	0.49% (.22–.77)	.17% (.06–.30)	.32% (.03–.64)	2.90 (1.12–9.29)
	4	1.21% (.72–1.79)	.41% (.23–.72)	.80% (.23–1.40)	2.93 (1.35–6.03)
	6	2.13% (1.30–3.11)	.83% (.51–1.29)	1.30% (.37–2.31)	2.57 (1.39–4.65)

Treatment-experienced with HIV					
		Risk for cardiovascular disease events			
Analysis	Time (years)	Switch to INSTI (95% CI)	Remained on Other ART (95% CI)	Risk Difference (95% CI)	Risk Ratio (95% CI)
Intention to treat	1	0.73% (.54–.95)	.47% (.43–.52)	.26% (.08–.47)	1.55 (1.16–2.00)
	2	1.25% (1.00–1.53)	.96% (.88–1.05)	.29% (.03–.58)	1.30 (1.03–1.62)
	4	2.01% (1.68–2.36)	1.95% (1.78—2.14)	.06% (−.29–.43)	1.03 (.86–1.22)
	6	2.69% (2.28–3.14)	2.90% (2.61–3.22)	−.22% (−.66–.25)	.93 (.78–1.09)
Per protocol	1	0.81% (.67–.96)	.45**%** (.41–.49)	.37% (.22–.53)	1.82 (1.47–2.23)
	2	1.43% (1.23–1.64)	.90% (.82–.99)	.52% (.30–.76)	1.58 (1.31–1.88)
	4	2.38% (2.14–2.67)	1.85% (1.66–2.11)	.53% (.15–.90)	1.29 (1.07–1.54)
	6	3.31% (2.95–3.65)	2.86% (2.46–3.36)	.45% (−.16–1.04)	1.16 (.95–1.41)

**INSTI** = integrase strand transfer inhibitor, **ART** = antiretroviral therapy, **CI** = confidence interval.

### Treatment-experienced Analysis

As of January 2012, 28 638 individuals had a suppressed HIV viral load on INSTI-free ART. After removing 691 individuals with a history of CVD, and 5026 with missing information on CVD history, we included 22 921 people with HIV (Figure 1*B*). Individuals could be included in multiple monthly trials if they fulfilled the inclusion criteria and were thus replicated (leading to 1 299 172 nonunique individuals). Overall, 28% of participants were female and 12% Black, and the median age at baseline was 49 years (IQR 41–56, Table 3).

**Table 3.** Baseline characteristics of the treatment-experienced population of nonunique people with HIV

Characteristic	Switched To INSTI N = 11'278	Remained on Other ART N = 1'287'894	Overall N = 1'299'172
Female	3009 (27%)	358'469 (28%)	361'478 (28%)
Median age, years (IQR)	50 (43–56)	49 (41–56)	49 (41–56)
Ethnicity			
White	7877 (70%)	909'424 (71%)	917'301 (71%)
Black	1170 (10%)	152'163 (12%)	153'333 (12%)
Other	346 (3.1%)	44'476 (3.5%)	44'822 (3.5%)
Unknown	1885 (17%)	181'831 (14%)	183'716 (14%)
Cohort region			
Western Europe	6439 (57%)	660'151 (51%)	666'590 (51%)
Southern Europe	1918 (17%)	236'073 (18%)	237'991 (18%)
Northern Europe / Australia	1897 (17%)	238'785 (19%)	240'682 (19%)
Eastern Europe	1024 (9.1%)	152'885 (12%)	153'909 (12%)
HIV transmission risk			
MSM	4812 (43%)	548'011 (43%)	552'823 (43%)
Person who injects drugs	1864 (17%)	174'735 (14%)	176'599 (14%)
Heterosexual	3943 (35%)	491'057 (38%)	495'000 (38%)
Other	280 (2.5%)	24'978 (1.9%)	25'258 (1.9%)
unknown	379 (3.4%)	49'113 (3.8%)	49'492 (3.8%)
CD4 cell count			
≥500 cells/µL	7936 (70%)	910'504 (71%)	918'440 (71%)
350–499 cells/µL	1956 (17%)	236'256 (18%)	238'212 (18%)
200–349 cells/µL	983 (8.7%)	112'058 (8.7%)	113'041 (8.7%)
<200 cells/µL	395 (3.5%)	28'622 (2.2%)	29'017 (2.2%)
missing	8 (<.1%)	454 (<.1%)	462 (<.1%)
Nadir CD4 cell count			
≥500 cells/µL	1010 (9.0%)	98'074 (7.6%)	99'084 (7.6%)
350–499 cells/µL	1552 (14%)	170'091 (13%)	171'643 (13%)
200–349 cells/µL	3745 (33%)	459'580 (36%)	463'325 (36%)
50–199 cells/µL	3446 (31%)	399'389 (31%)	402'835 (31%)
<50 cells/µL	1520 (13%)	160'604 (12%)	162'124 (12%)
Unknown	5 (<0.1%)	156 (<0.1%)	161 (<0.1%)
Receiving ABC-based ART	4607 (41%)	300'399 (23%)	305'006 (23%)
Receiving TAF-based ART	2951 (26%)	135'995 (11%)	138'946 (11%)
Year of ART start			
Before 2001	4083 (36%)	451'137 (35%)	455'220 (35%)
2001–2010	3570 (32%)	451'807 (35%)	455'377 (35%)
After 2010	3625 (32%)	384'950 (30%)	388'575 (30%)
Baseline year of trial			
Before 2015	2289 (20%)	563'133 (44%)	565'422 (44%)
2015—2016	4386 (39%)	314'885 (24%)	319'271 (25%)
After 2016	4603 (41%)	409'876 (32%)	414'479 (32%)
Renal function (eGFR)			
≥90 mL/min	5802 (51%)	741'184 (58%)	746'986 (57%)
60–89 mL	4150 (37%)	441'102 (34%)	445'252 (34%)
30–59 mL	681 (6.0%)	45'981 (3.6%)	46'662 (3.6%)
<30 mL/min	58 (0.5%)	3214 (0.2%)	3272 (0.3%)
Unknown	587 (5.2%)	56'413 (4.4%)	57'000 (4.4%)
Diabetes			
No	9769 (87%)	1'140'059 (89%)	1'149'828 (89%)
Yes	730 (6.5%)	71'230 (5.5%)	71'960 (5.5%)
Unknown	779 (6.9%)	76'605 (5.9%)	77'384 (6.0%)
BMI category			
Underweight (<18.5 kg/m^2^)	423 (3.8%)	38'088 (3.0%)	38'511 (3.0%)
Normal (18.5—24.9 kg/m^2^)	5361 (48%)	602'515 (47%)	607'876 (47%)
Overweight (25.0—29.9 kg/m^2^)	2992 (27%)	344'832 (27%)	347'824 (27%)
Obese (≥30 kg/m^2^)	1167 (10%)	125'450 (9.7%)	126'617 (9.7%)
Unknown	1335 (12%)	177'009 (14%)	178'344 (14%)
Arterial hypertension			
No	6651 (59%)	780'215 (61%)	786'866 (61%)
Yes	3661 (32%)	387'051 (30%)	390'712 (30%)
Unknown	966 (8.6%)	120'628 (9.4%)	121'594 (9.4%)
Smoking status			
Never	3180 (28%)	399'745 (31%)	402'925 (31%)
Current	3776 (33%)	420'886 (33%)	424'662 (33%)
Previous	2239 (20%)	251'375 (20%)	253'614 (20%)
Unknown	2083 (18%)	215'888 (17%)	217'971 (17%)
Total cholesterol			
<4.1 mmol/L	2007 (18%)	217'683 (17%)	219'690 (17%)
4.1–5.19 mmol/L	4072 (36%)	483'804 (38%)	487'876 (38%)
5.2–6.19 mmol/L	2905 (26%)	349'907 (27%)	352'812 (27%)
6.2–7.19 mmol/l	1140 (10%)	128'498 (10.0%)	129'638 (10.0%)
≥7.2 mmol/L	408 (3.6%)	40'344 (3.1%)	40'752 (3.1%)
Unknown	746 (6.6%)	67'658 (5.3%)	68'404 (5.3%)
HDL			
<0.9 mmol/L	1234 (11%)	113'818 (8.8%)	115'052 (8.9%)
0.9–1.19 mmol/L	2989 (27%)	328'643 (26%)	331'632 (26%)
1.2–1.29 mmol/L	999 (8.9%)	119'970 (9.3%)	120'969 (9.3%)
1.3–1.59 mmol/l	2389 (21%)	277'723 (22%)	280'112 (22%)
≥1.6 mmol/L	2587 (23%)	319'915 (25%)	322'502 (25%)
Unknown	1080 (9.6%)	127'825 (9.9%)	128'905 (9.9%)
Receiving lipid-lowering treatment	1046 (9.3%)	104'843 (8.1%)	105'889 (8.2%)
Receiving antiplatelet drugs	828 (7.3%)	74'040 (5.7%)	74'868 (5.8%)

IQR = interquartile range, INSTI = integrase strand transfer inhibitor, ART = antiretroviral therapy, N = number, MSM = men who have sex with men, ABC = abacavir, TAF = tenofovir alafenamide, eGFR = estimated glomerular filtration rate, CV = cardiovascular, BMI = body mass index, HDL = high-density lipoprotein.

Baseline characteristics between individuals who switched to INSTI-based and those who remained on other ART were similar, including smoking status, presence of arterial hypertension, and diabetes. However, individuals who switched to INSTI-based ART were more likely to have an eGFR <60 mL/min and were less likely to be from an Eastern European cohort. Among individuals who switched to INSTIs, the majority received dolutegravir (n = 6558, 58%), followed by elvitegravir (2037, 18.1%), raltegravir (1819, 16.1%), bictegravir (n = 853, 7.6%), and cabotegravir (11, 0.1%). Among individuals who stayed on non-INSTI ART, most common combinations included efavirenz (23.3%), followed by darunavir (16.8%), rilpivirine (14.3%), nevirapine (13.8%), atazanavir (9.7%), and lopinavir (5.3%). Covariate balance before and after inverse-probability weighting is shown in Supplementary Figure 4.

Individuals contributed to a median of 57 trials (IQR 32–94) and were followed for a median of 37 months (IQR 18–63). During this follow-up, 800 unique CVD events occurred: 311 myocardial infarctions (38.9%), 256 strokes (32%), and 233 invasive cardiovascular procedures (29.1%). Individuals who switched to INSTI-based ART experienced 286 CVD events, compared to 19 596 nonunique events during the sequential trials among individuals who remained on other ART (Supplementary Table 5). In the ITT analysis, adjusted risk ratios for CVD events among individuals who switched to an INSTI compared to those who remained on other ART were 1.55 (95% CI 1.16–2.00) at 1 year, 1.30 (1.03–1.62) at 2 years, and 0.93 (95% CI .78–1.09) at 6 years (Figure 2*B*). Absolute risk differences reached 0.29 percentage points (95% CI .03–.58) at 2 years and subsequently decreased. Per-protocol analyses confirmed these findings (Table 2, Supplementary Figure 3B).

### Subgroup and Sensitivity Analyses

The impact of switching to INSTIs versus remaining on other ART among treatment-experienced individuals was similar across age groups (*P*-value for interaction=.19) and did not differ by sex/gender (*P* = .96) or ABC use at baseline (*P* = .72, Supplementary Figure 5). Intention-to-treat results were similar when estimated using parametric g-computation instead of inverse probability of treatment weighting (Supplementary Table 6). Results were also consistent after restricting to sequential trials starting after January 2017 (23 939 individuals). When limiting the analysis to cohorts sharing data on all of their participants with RESPOND (4 cohorts, 6887 participants, 233 events), the point estimates were attenuated and no longer statistically significant, with risk ratios of 1.15 (95% CI .64–1.63) after Year 1 and 0.97 (.57–1.33) after Year 2 (Supplementary Table 7, Supplementary Figure 6).

## DISCUSSION

As part of our pharmacovigilance efforts, we reexamined a safety signal suggesting an increased CVD risk with INSTI use in RESPOND. Using a multi-cohort target trial emulation, up to 2 additional years of follow-up per participant and 700 additional participants compared to our initial analysis, we found that treatment-experienced individuals switching to INSTIs had a significantly higher CVD risk during the first 2 years—ranging from 41% (ITT) to 74% (PP) relative increase—compared to those remaining on other ART. Among treatment-naïve individuals, CVD risk is also higher in people initiating INSTIs compared to those receiving other ART and continued to diverge over time, although low event numbers limit our ability to draw firm conclusions. Importantly, absolute risk differences remained small throughout, never exceeding 2.30 percentage points. These findings suggest that while INSTIs are generally well tolerated, the potential for an increased CVD risk remains a concern.

Our findings confirm and extend the results of our previous study, which showed a sharp early increase in CVD after INSTI initiation [12]. Because this pattern differs from conventional CVD pathophysiology, methodological concerns were raised [24]. In this target trial emulation, we observed a similar pattern of comparable magnitude among treatment-experienced individuals—the majority of participants in the original study—supporting the robustness of our initial observation [14]. These findings remained consistent when using a different causal inference estimator. Another consideration was potential selection bias due to RESPOND’s stratified selection of cohort participants by INSTI versus non-INSTI use. In the sensitivity analysis restricted to cohorts that provide data on all their cohort participants, the association between switching to INSTI-based ART and CVD risk was attenuated and no longer statistically significant. This restricted analysis included substantially fewer participants and CVD events, resulting in wider CI and reduced precision. The attenuation may also reflect differences in participant characteristics, treatment patterns, calendar period, or cohort-level data structures between the four cohorts included in this sensitivity analysis and the broader RESPOND population. These findings suggest some sensitivity to cohort inclusion. However, the primary results were compatible with those from the HIV-CAUSAL Collaboration, in which participant selection was not stratified by INSTI use, providing reassurance that the observed short-term association is unlikely to be explained solely by RESPOND's stratified sampling approach [18].

Other target trial emulations assessing the effect of INSTIs on CVD risk among treatment-experienced individuals include HIV-CAUSAL, which found no difference in the 4-year CVD risks between individuals who switched to INSTIs and those remaining on other ART [18]. However, less attention was given to the dynamics in the first 2 years, where the safety signal was observed. Comparing graphs from the present study and HIV-CAUSAL, a similar pattern with an early increase in CVD risk within the first 2 years was seen. Confidence intervals overlapped in HIV Causal due to a lower number of events. Further studies among treatment-experienced individuals are needed to confirm and understand the mechanisms behind these findings.

Our results among treatment-naïve individuals indicated an increased CVD risk after starting INSTI compared to other ART, which increased over time. Such a gradual increase is compatible with the conventional development of atherosclerosis, potentially accelerated due to the impact of INSTIs on the development of obesity, arterial hypertension, and diabetes [13, 25]. This finding contrasts with those from the SHCS and HIV-CAUSAL, where no increase in CVD risk was observed after starting INSTI-based ART [17, 18]. While the two prior target trial emulations have not shown significant differences, two US-based studies using claims data reported an increase in CVD risk among treatment-naïve individuals starting INSTIs compared to those initiating other ART components, like our findings. In the study by Parra-Rodriguez et al., individuals starting INSTIs or PIs had a higher risk of CVD events compared to those initiating NNRTIs [15]. Similarly, Rebeiro et al. found that the elevated CVD risk with INSTI use was driven by myocardial infarction and congestive heart failure, rather than stroke [16]. Differences in study design and population characteristics likely contribute to the divergent findings. However, due to the low number of events in both the ITT and PP analyses in our analysis, this finding should be interpreted with caution.

To date, no convincing causal mechanism has been identified yet explaining the rapid early increase in CVD risk observed among treatment-experienced people with HIV. A recent untargeted metabolomic study from the SHCS identified drug-specific metabolic perturbations that may inform pathways linking INSTI use to CVD risk [26]. In addition, preliminary data suggest that some INSTIs differentially affect adhesion molecule expression involved in vascular, platelet, and leukocyte interactions [27].

For this analysis, we leveraged systematic ascertainment, stringent case reporting and validation of CVD events, and a large population with extended follow-up within RESPOND. Applying a target trial framework also reduced potential bias from study design. However, our study has limitations. First, the observed rapid risk increase—diminishing over time—is compatible with confounding by indication and other noncausal explanations. Although we adjusted our analysis for many known CVD risk factors, residual confounding, channeling of higher-risk individuals to INSTI-based regimens, and differential healthcare contact after treatment switching cannot be excluded. Second, selection bias may have influenced our results. However, with very similar patterns seen in HIV-CAUSAL collecting data on all participants without selection, our findings are unlikely to be driven only by this. Finally, as in our initial analysis, we focused on INSTIs as a class. However, given that dolutegravir was the predominant agent (55.1% of treatment-naïve and 58% of treatment-experienced participants), findings may reflect dolutegravir exposure more than a uniform class effect. Further studies with sufficient power to examine both within-class differences and the differential cardiovascular risk of individual comparator regimens will be needed to disentangle these contributions.

In conclusion, using a target trial framework designed to reduce bias, we confirmed the findings of an increase in relative CVD risk associated with INSTI use, with differing patterns among treatment-naïve and treatment-experienced individuals. However, the absolute risk difference remained small, and given the many benefits of INSTI-based ART which may outweigh the transiently elevated risk of CVD, INSTIs should remain recommended by HIV treatment guidelines. Having addressed key methodological concerns, the next phase of pharmacovigilance should focus on evaluating use of individual INSTIs and conducting studies exploring potential underlying mechanisms.

## Supplementary Material

ofag490_Supplementary_Data

## Supplementary Data

Supplementary materials are available at *Open Forum Infectious Diseases* online. Consisting of data provided by the authors to benefit the reader, the posted materials are not copyedited and are the sole responsibility of the authors, so questions or comments should be addressed to the corresponding author.
