# Supplementary material for: Cardiovascular Disease Risk with Integrase Inhibitor-based Antiretroviral Therapy: A Causal Inference Analysis in RESPOND
Source: Open Forum Infect Dis. 2026 Aug 6;13(8):ofag490. doi: 10.1093/ofid/ofag490 (PMC13479176; doi:10.1093/ofid/ofag490)
Supplement: Supplementary file 1 [file ofag490_supplementary_data.pdf]

Supplementary Material

Evaluating the impact of integrase inhibitors on cardiovascular disease events:

A causal inference analysis in RESPOND

Bernard Surial[1*], Bastian Neesgaard[2*], Frédérique Chammartin[3], Lene Ryom[1,4,5], Kathy Petoumenos[6], Joanne Reekie[2], Roger Paredes[7], Robert Zangerle[8], Colette Smith[9], Charlotte Martin[10], Ferdinand Wit[11], Nadine Jaschinski[2], Cristina Mussini[12], Antonella d'Arminio Monforte[13], Antonella Castagna[13], Christina Carlander[14], Larysa Hetman[15], Elena Sohani[8], Clara Lehmann[16,17], Sean R Hosein[18], Jean Andre Van Wyk[19], Emiliano Bissio[20], Jens Lundgren[2], Andri Rauch[1], Huldrych F. Günthard[21,22], Gilles Wandeler[1,23], Lauren Greenberg[2]

Table of content

Table S1: Key protocol elements of the treatment-naïve analysis 2
Table S2: Key protocol elements of the treatment-experienced analysis 3
Figure S1: Description of the sequential nested trial approach 4
Supplementary note: Time zero alignment and handling of early events 5
Table S3: Diagnostics of the weights used for the main analyses 5
Figure S2: Covariate balance in the treatment-naïve population before and after weighting 6
Table S4: Individuals at risk and cumulative cardiovascular disease events in the treatment-naïve analysis 6
Figure S3: Adjusted cumulative risk of developing cardiovascular disease events (per-protocol analysis) 7
Figure S4: Covariate balance in the treatment-experienced population before and after weighting 8
Table S5: Individuals at risk and cumulative cardiovascular disease events in the treatment-experienced analysis 8
Figure S5: Subgroup analyses among treatment-experienced individuals with HIV (intention to treat analysis) 9
Figure S6: Sensitivity Analyses 11

Supplementary Material

Table S1: Key protocol elements of the treatment-naïve analysis

	**Target trial**	**Emulated trial**
**Eligibility criteria**	- HIV infection<br>- Aged ≥18 years at baseline<br>- ART-naïve<br>- Detectable HIV viral load (≥200 cp/mL)	- HIV infection<br>- Aged ≥18 years at baseline<br>- ART-naïve after 01/2012 (when INSTIs became widely available in Europe)<br>- Detectable HIV viral load (≥200 cp/mL)<br>- Have a CD4 count and viral load measurement either 1 year before or within 12 weeks after ART start<br><br>The following individuals will be excluded:<br>- Individuals who started maraviroc or enfuvirtide (as they may not have been treatment-naïve)<br>- Individuals who started an unknown ART regimen (e.g., participants in blinded randomized trials)<br>- Missing data on gender/sex
**Treatment strategies**	1. Starting INSTI-based ART<br>2. Starting non-INSTI based ART	Same as target trial
**Baseline (Time-0)**	Baseline will be defined as the start date of the initial ART regimen.	Same as target trial
**Treatment assignment**	Patients are randomly assigned and are aware of the strategy they are assigned to	Randomization is assumed conditional on baseline covariates: age, gender/sex, race/ethnicity, local cohort, HIV transmission risk, nadir and baseline CD4 cell counts, HIV viral load, year of ART start, history of AIDS-defining illness, body mass index, diabetes, arterial hypertension, estimated glomerular filtration rate, history of CVD, smoking status, blood lipid levels, and whether initial ART included tenofovir alafenamide or abacavir.
**Censoring**	In the intention-to-treat analysis: Individuals will be followed from baseline to the first of CVD event, death, loss to follow-up, or the latest cohort visit (defined as the latest of final ART start, final VL measurement, or final CD4 measurement).<br>In the per-protocol analysis: Individuals who stopped the assigned strategy will be censored at that time (artificial censoring).	Same as target trial. In addition, individuals without HIV-RNA measurement for 18 months or more will be considered “lost to follow-up”.
**Outcomes**	Composite endpoint consisting of the first of fatal and non-fatal myocardial infarctions, strokes, and invasive cardiovascular procedures	Same as target trial

Table S2: Key protocol elements of the treatment-experienced analysis

	**Target trial**	**Emulated trial**
**Eligibility criteria**	- HIV infection<br>- Aged ≥18 years at baseline<br>- On INSTI-free ART after 01/2012<br>- No prior exposure to INSTI<br>- Suppressed HIV viral load (<200 cp/mL) at baseline	- HIV infection<br>- Aged ≥18 years at baseline<br>- On INSTI-free ART after 01/2012<br>- No prior exposure to INSTI<br>- Suppressed HIV viral load (<200 cp/mL) at baseline<br>- Have a CD4 count and viral load measurement either 1 year before or within 12 weeks after baseline<br><br>The following individuals will be excluded:<br>- Individuals who started maraviroc or enfuvirtide<br>- Individuals who started an unknown ART regimen<br>- Missing data on gender/sex<br>- Individuals with a history of cardiovascular disease (CVD)
**Treatment strategies**	1. Switching to INSTI-based ART<br>2. Remaining on non-INSTI based ART (note that switching from non-INSTI based ART to a different ART combination without INSTI is allowed for this strategy)	Same as target trial
**Baseline (Time-0)**	At randomization	Sequential trial approach: A new trial will be emulated at the beginning of every month, starting in 01/2012 until the month before database closure.
**Treatment assignment**	Patients are randomly assigned and are aware of the strategy they are assigned to	Randomization is assumed conditional on baseline covariates: age, gender/sex, race/ethnicity, local cohort, HIV transmission risk, nadir and baseline CD4 cell counts, year of ART start, history of AIDS-defining illness, body mass index, diabetes, arterial hypertension, estimated glomerular filtration rate, smoking status, blood lipid levels, and whether initial ART included tenofovir alafenamide or abacavir.
**Censoring**	In the intention-to-treat analysis: Individuals will be followed from baseline to the first of CVD event, death, loss to follow-up, or the latest cohort visit (defined as the latest of final ART start, final VL measurement, or final CD4 measurement).<br>In the per-protocol analysis: Individuals who stopped the assigned strategy will be censored at that time (artificial censoring).	Same as target trial. In addition, individuals without HIV-RNA measurement for 18 months or more will be considered “lost to follow-up”.
**Outcomes**	Same as treatment-naïve analysis.	Same as target trial

A Illustration of two treatment-experienced patients in the cohort database

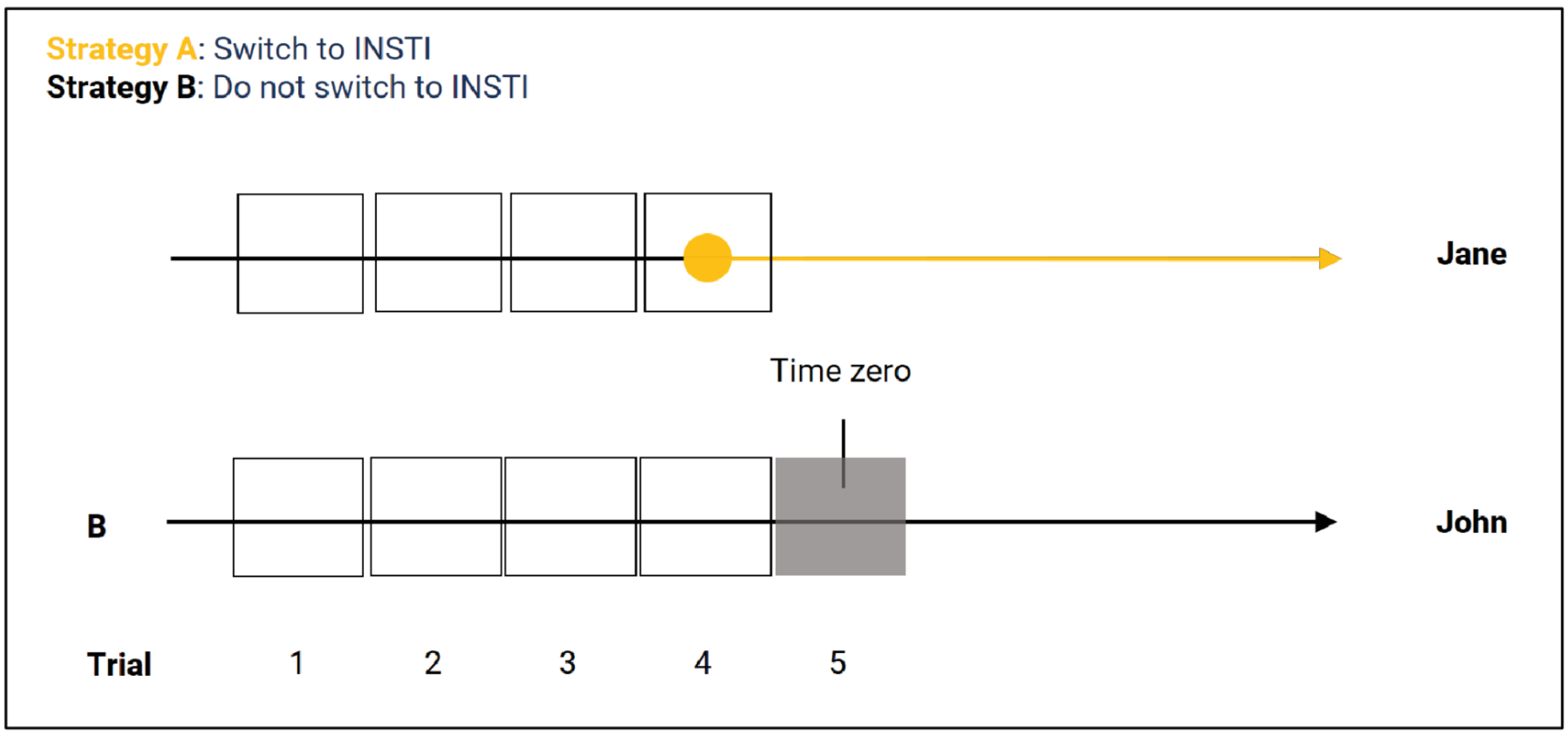

B Description of the dataset using a sequential trial approach

ID	Trial	Start	Stop (PP)	Stop (ITT)	Strategy
JANE	1	January	April	December	**B – no switch**
JOHN	1	January	September	September	**B – no switch**
JANE	2	February	April	December	**B – no switch**
JOHN	2	February	September	September	**B – no switch**
JANE	3	March	April	December	**B – no switch**
JOHN	3	March	September	September	**B – no switch**
JANE	4	April	December	December	**A – switch**
JOHN	4	April	September	September	**B – no switch**
JOHN	5	May	September	September	**B – no switch**
JOHN	6	June	September	September	**B – no switch**

Figure S1: Description of the sequential nested trial approach

(**A**) Jane is a treatment-experienced cohort participant who switched to INSTI in April, while John remains on non-INSTI based ART throughout the cohort follow-up. Follow-up stops for administrative reasons in December for Jane and in September for John (not shown in graph). To determine a sensible baseline for both individuals, we emulate a sequence of trials every month, starting in January (trial 1). Baseline (or time zero) will be reestablished for every trial independently, and eligibility will be assessed at each trial baseline. Jane and John are both eligible for trials 1-4. Jane will be categorized into strategy B for trials 1-3, and into strategy A for trial 4. As Jane is no longer INSTI-naïve in trial 5, she is not eligible anymore and hence not included in this trial. John will be eligible for all trials 1-5, and therefore categorized as such. (**B**) The table outlines the resulting dataset with the start and stop date for each trial. Whereas follow-up continues until the event of follow-up in the intention-to-treat (ITT) analysis, individuals will be artificially censored once they deviate from the initial strategy in the per-protocol (PP) analysis. Artificial censoring occurred for Jane in trials 1-3 when she switched to INSTI in April. Note that this approach leads to a replication of individuals as long as they follow strategy B.

Supplementary note: Time zero alignment and handling of early events

Eligibility was assessed at each monthly trial start without a formal eligibility window or grace period. The sole exception was CD4 and HIV viral load measurements, which were permitted within 12 weeks after baseline; however, post-baseline measurements accounted for only 0.8% of instances and all occurred within 4 weeks. Treatment assignment was determined by the closest trial start date.

Table S3: Diagnostics of the weights used for the main analyses

Population	Primary weight	N	Min	Median	Mean	P95	P99	Max	ESS	ESS/N
Treatment-naïve (intention to treat)	Stabilized IPTW, truncated at 99th percentile	7111	0.491	0.647	0.982	2.698	6.870	6.874	3’548	0.499
Treatment-naïve (per protocol)	IPTW × IPCW, truncated at 99th percentile	330’750	0.229	0.604	0.958	2.780	7.349	7.349	151’277	0.457
Treatment-experienced (intention to treat)	Stabilized IPTW, truncated at 99th percentile	1’299’172	0.990	1.000	1.000	1.020	1.030	1.030	1’299’092	1.000
Treatment-experienced (per protocol)	Cumulative IPTW, truncated at 99th percentile	54’352’116	1.000	1.196	*	3.032	23.074	23.074	18’741’256	0.345

IPTW =inverse probability of treatment weighting; **IPCW** = inverse probability of censoring weighting; **ESS** = Kish effective sample size. **N** denotes the number of observations/person-time units contributing to the weighted analysis. **P95** and **P99** denote the 95th and 99th percentiles of the weight distribution. For the treatment-experienced per-protocol analysis, summaries refer to cumulative inverse probability weights among fit-eligible person-time after sequential trial expansion.

*The mean p99-truncated cumulative weight was not retained for the treatment-experienced per-protocol analysis; median and p95 are unchanged by p99 truncation.

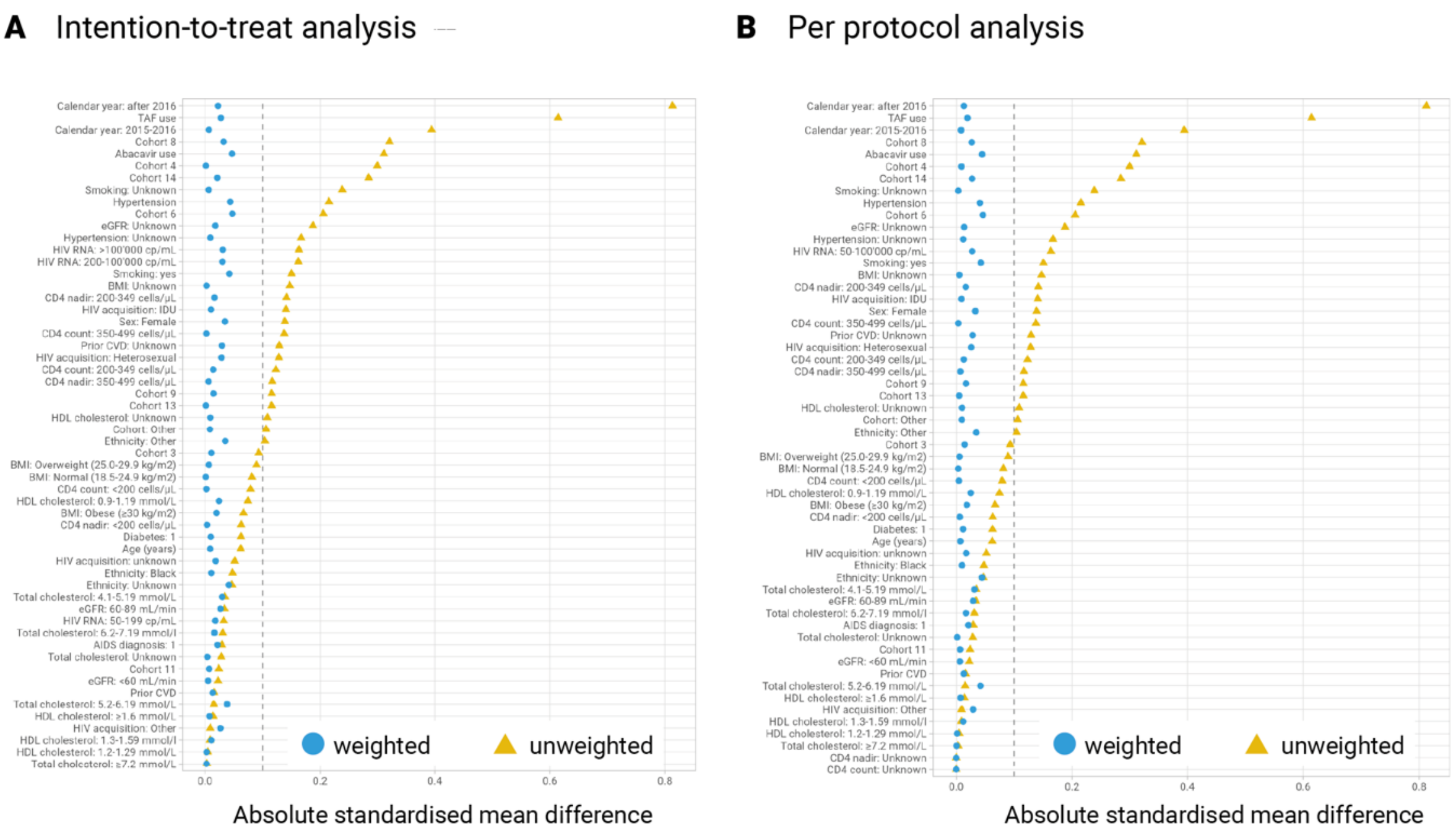

Figure S2: Covariate balance in the treatment-naïve population before and after weighting
After applying inverse probability of treatment weighting (**blue circles**), covariates (expressed as absolute standardised mean differences) were well balanced between individuals who started integrase inhibitors compared to those who started other antiretroviral therapy. **Panel A** shows the intention-to-treat population; **panel B** shows the per protocol population. The dashed vertical line denotes a standardised mean difference of 0.1, a common threshold used to determine covariate balance.

Table S4: Individuals at risk and cumulative cardiovascular disease events in the treatment-naïve analysis

	Started other ART			Started INSTI based ART		
**Years**	**Individuals at risk (proportion)**	**Cumulative number of CVD events**	**Cumulative incidence of CVD events**	**Individuals at risk (proportion)**	**Cumulative number of CVD events**	**Cumulative incidence of CVD events**
0	3757 (100%)	–	–	3354 (100%)	–	–
1	3530 (94%)	6	0·16%	3063 (91%)	9	0·28%
2	3330 (89%)	11	0·31%	2698 (80%)	20	0·66%
3	3103 (83%)	19	0·56%	2242 (67%)	25	0·86%
4	2833 (75%)	25	0·76%	1785 (53%)	29	1·06%
5	2524 (67%)	33	1·06%	1244 (37%)	39	1·72%
6	2109 (56%)	35	1·15%	728 (22%)	44	2·31%
7	1585 (42%)	41	1·45%	324 (10%)	48	2·96%
8	939 (25%)	43	1·62%	123 (4%)	50	4·19%
9	382 (10%)	45	1·92%	33 (1%)	50	4·19%
10	1 (0%)	45	1·92%	0 (0%)	50	4·19%

Based on intention-to-treat population.
ART = antiretroviral therapy, **INSTI** = integrase strand transfer inhibitor, **CVD** = cardiovascular disease.

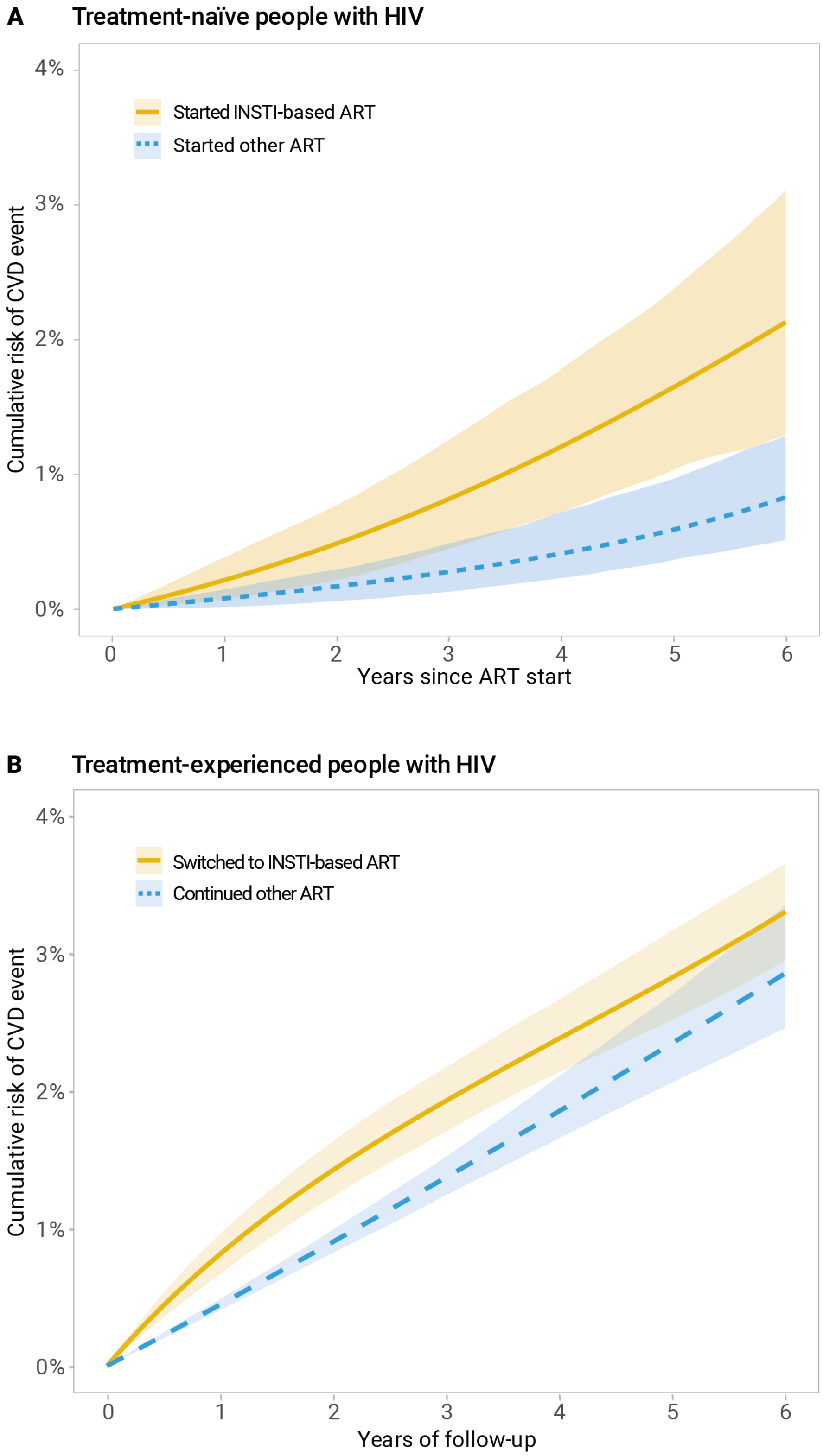

Figure S3: Adjusted cumulative risk of developing cardiovascular disease events (per-protocol analysis)

The cumulative risk (*line*) of developing cardiovascular disease (**CVD**) events and corresponding 95% confidence intervals (*shaded area*) are shown for 7111 treatment naïve (**panel A**) and 22'921 treatment–experienced (**panel B**) people with HIV. CVD events include myocardial infarction, stroke, and procedures on arteries. Results are shown for the per-protocol analysis, and confidence intervals were calculated using bootstrapping with 500 samples.
INSTI = integrase strand transfer inhibitor, **ART** = antiretroviral therapy.

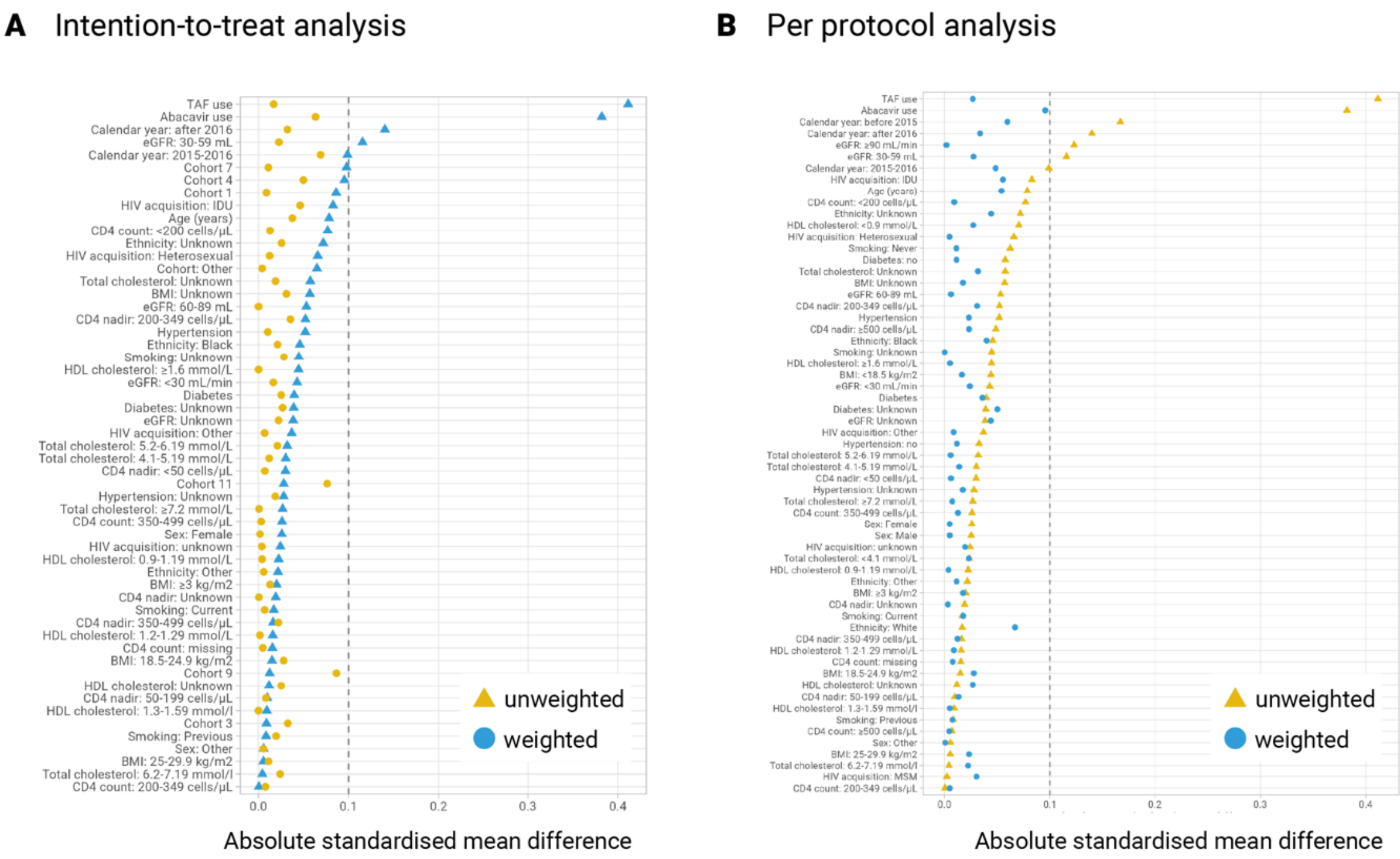

Figure S4: Covariate balance in the treatment-experienced population before and after weighting
After applying inverse probability of treatment weighting (**blue circles**), covariates (expressed as absolute standardised mean differences) were well balanced between individuals who switched to integrase inhibitors compared to those who remained on other antiretroviral therapy. **Panel A** shows the intention-to-treat population; **panel B** shows the per protocol population. The dashed vertical line denotes a standardised mean difference of 0.1, a common threshold used to determine covariate balance.

Table S5: Individuals at risk and cumulative cardiovascular disease events in the treatment-experienced analysis

	Continued other ART			Switched to INSTI-based ART		
**Years**	**Individuals at risk (proportion)**	**Cumulative number of CVD events**	**Cumulative incidence of CVD events**	**Individuals at risk (proportion)**	**Cumulative number of CVD events**	**Cumulative incidence of CVD events**
0	1’287’894 (100%)	–	–	11’278 (100%)	–	–
1	1’079’983 (84%)	5290	0·44%	10’275 (91%)	87	0·80%
2	856’668 (67%)	9506	0·88%	8984 (80%)	149	1·45%
3	657’528 (51%)	12’702	1·30%	7846 (70%)	192	1·95%
4	488’630 (38%)	15’023	1·71%	6349 (56%)	231	2·48%
5	351’625 (27%)	16’703	2·11%	4576 (41%)	250	2·82%
6	241’350 (19%)	17’940	2·53%	2714 (24%)	265	3·26%
7	153’431 (12%)	18’780	2·95%	1150 (10%)	274	3·74%
8	83’635 (6%)	19’314	3·41%	439 (4%)	281	4·54%
9	29’860 (2%)	19’560	3·84%	139 (1%)	286	6·17%
10	199 (0%)	19’596	4·06%	0 (0%)	286	6·17%

Based on intention-to-treat population. Note that due to the sequential nested trial approach, individuals at risk and CVD events are non-unique (see **Figure S1**).
ART = antiretroviral therapy, **INSTI** = integrase strand transfer inhibitor, **CVD** = cardiovascular disease.

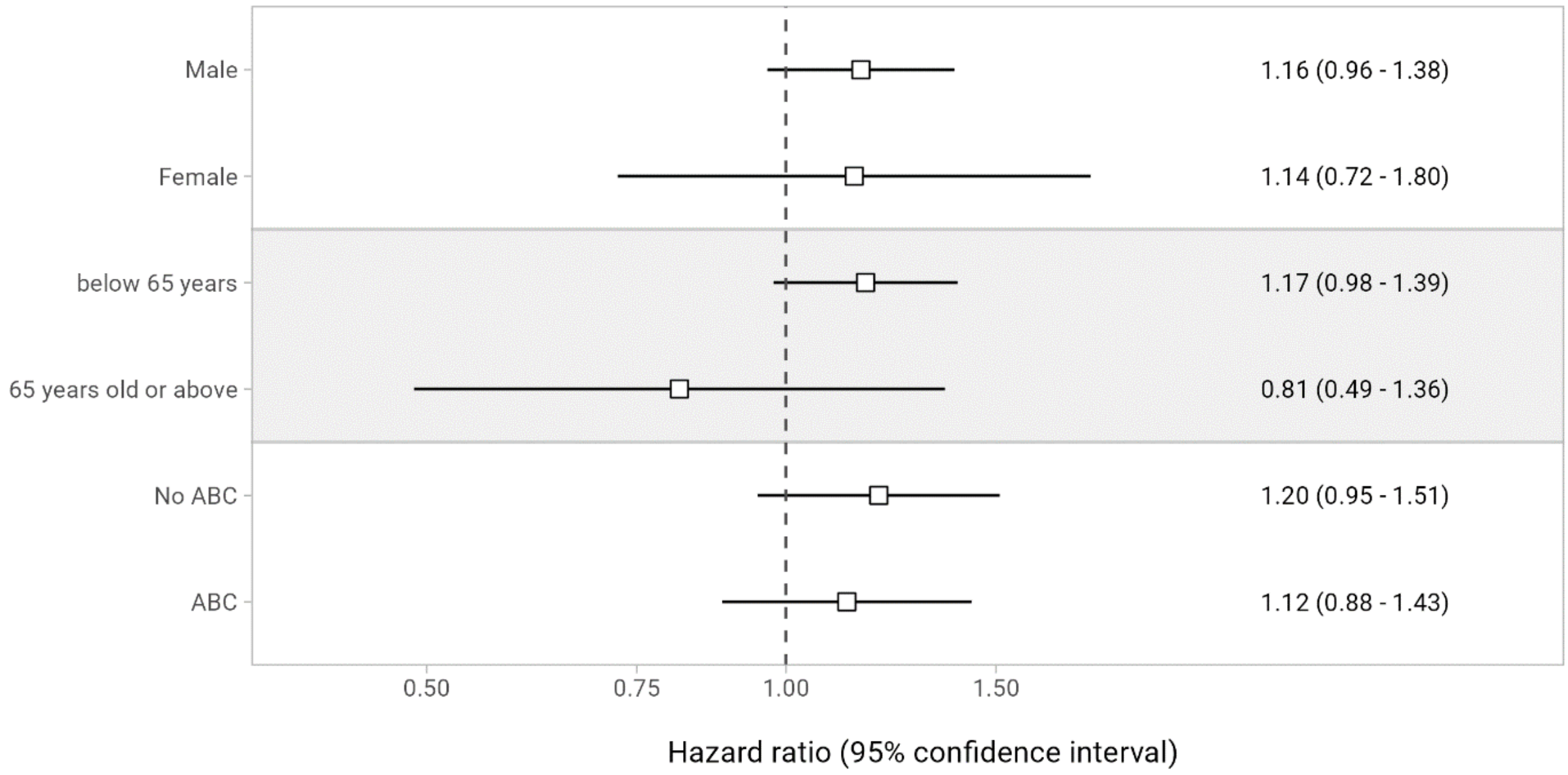

Figure S5: Subgroup analyses among treatment-experienced individuals with HIV (intention to treat analysis)

The forest plot shows hazard ratios (and 95% confidence intervals) for experiencing a cardiovascular disease event with integrase inhibitors compared to other antiretroviral therapies, stratified by gender/sex, age and whether the baseline antiretroviral regimen included abacavir (**ABC**). Confidence intervals are calculated using robust standard errors.

Table S6: Intention-to-treat analyses using parametric g-computation

Analysis	Time (years)	Risk for cardiovascular disease events: Started INSTI (95% CI)	Started other ART (95% CI)	Risk Difference (95% CI)	Risk Ratio (95% CI)
Treatment-naïve	1	0.29% (0.15 – 0.45)	0.15% (0.07 – 0.26)	0.14% (-0.04 – 0.32)	1.92 (0.82 – 4.31)
	2	0.57% (0.33 – 0.84)	0.31% (0.17 – 0.49)	0.26% (-0.07 – 0.56)	1.83 (0.83 – 3.68)
	4	1.19% (0.79 – 1.68)	0.71% (0.44 – 1.08)	0.48% (-0.08 – 1.00)	1.68 (0.92 – 3.22)
	6	1.98% (1.31 – 2.62)	1.16% (0.80 – 1.69)	0.82% (-0.02 – 1.66)	1.71 (0.99 – 3.04)
Treatment-experienced	1	0.53% (0.42 – 0.64)	0.37% (0.34 – 0.40)	0.15% (0.07 – 0.27)	1.41 (1.19 – 1.71)
	2	0.96% (0.80 – 1.14)	0.76% (0.69 – 0.82)	0.20% (0.05 – 0.38)	1.26 (1.07 – 1.52)
	4	1.62% (1.37 – 1.91)	1.49% (1.33 – 1.67)	0.13% (-0.10 – 0.41)	1.09 (0.93 – 1.29)
	6	2.10% (1.63 – 2.64)	2.06% (1.82 – 2.40)	0.04% (-0.44 – 0.59)	1.02 (0.79 – 1.28)

INSTI = integrase strand transfer inhibitor, **ART** = antiretroviral therapy, **CI** = confidence interval.

Table S7: Sensitivity analyses among treatment-experienced individuals with HIV (intention to treat analysis)

Treatment–experienced with HIV: Followed in cohorts that share all cohort data with RESPOND (n = 6887)

Analysis	Time (years)	Risk for cardiovascular disease events: Switch to INSTI (95% CI)	Remained on Other ART (95% CI)	Risk Difference (95% CI)	Risk Ratio (95% CI)
Intention to treat	1	0·51% (0·28 – 0·71)	0·44% (0·35 – 0·52)	0·07% (-0·16 – 0·27)	1·15 (0·64 – 1·63)
	2	0·87% (0·54 – 1·21)	0·90% (0·73 – 1·06)	-0·03% (-0·39 – 0·30)	0·97 (0·57 – 1·33)
	4	1·42% (0·92 – 2·01)	1·67% (1·33 – 2·16)	-0·25% (-0·84 – 0·29)	0·85 (0·55 – 1·17)
	6	2·08% (1·19 – 3·71)	2·13% (1·57 – 2·96)	-0·05% (-1·18 – 1·39)	0·98 (0·48 – 1·66)

Treatment–experienced with HIV: Follow-up restricted to after January 2017 (n = 23'939)

Analysis	Time (years)	Risk for cardiovascular disease events: Switch to INSTI (95% CI)	Remained on Other ART (95% CI)	Risk Difference (95% CI)	Risk Ratio (95% CI)
Intention to treat	1	0·58% (0·38 – 0·79)	0·41% (0·33 – 0·47)	0·17% (-0·04 – 0·40)	1·41 (0·92 – 2·07)
	2	1·18% (0·84 – 1·59)	0·85% (0·69 – 1·03)	0·33% (0·02 – 0·74)	1·38 (1·04 – 1·93)
	4	2·19% (1·34 – 2·61)	1·78% (1·36 – 2·68)	0·41% (-0·56 – 0·70)	1·23 (0·78 – 1·42)

INSTI = integrase strand transfer inhibitor, **ART** = antiretroviral therapy, **CI** = confidence interval.

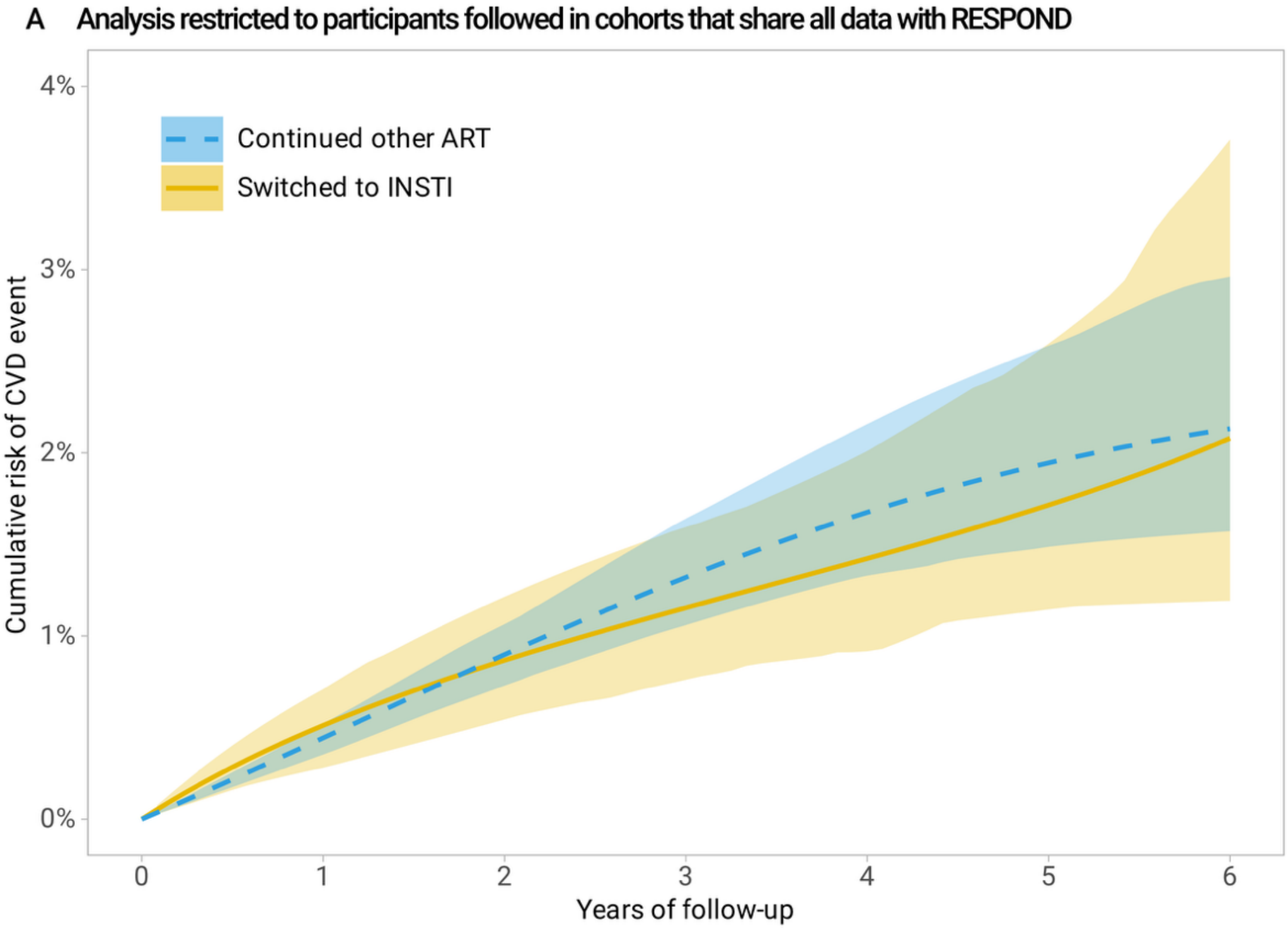

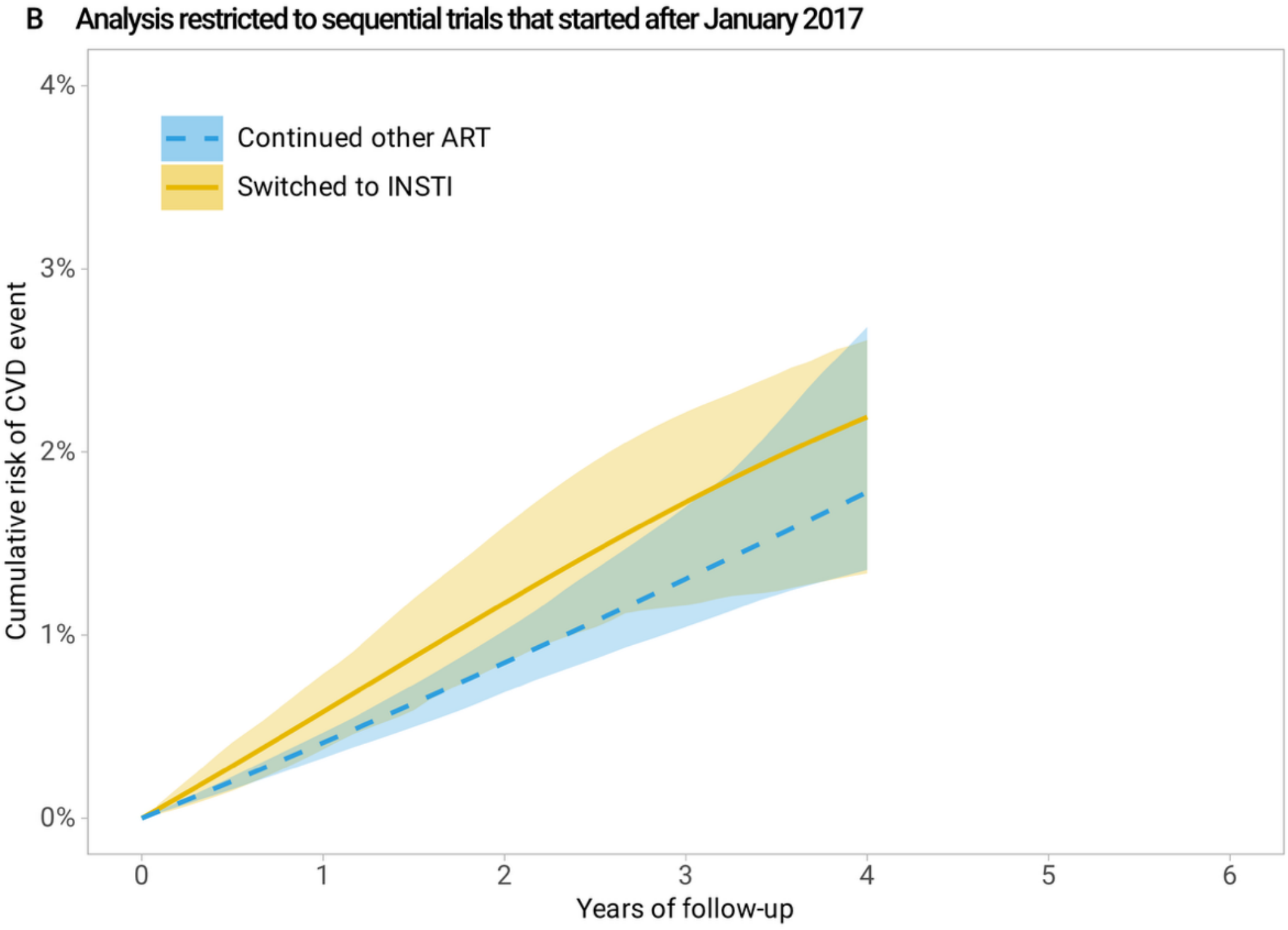

Figure S6: Sensitivity Analyses

Panel A shows adjusted cumulative incidences of cardiovascular disease (**CVD**) events among individuals who are followed in cohorts that share all data with RESPOND (n = 6887). **Panel B** shows cumulative incidence curves in the full population, but restricted to trials that started after January 2017, when data collection in RESPOND became prospective, and second-generation integrase inhibitors (**INSTI**) were more widely used. Confidence intervals were calculated using 200 bootstrap samples. **ART** = antiretroviral therapy.
